# The role of chemotherapy in patients with stage IB gastric adenocarcinoma: a real-world competing risk analysis

**DOI:** 10.1186/s12957-022-02591-5

**Published:** 2022-04-17

**Authors:** Yangyang Xie, Danwei Du, Xue Song, Xiaowen Li, Zhongkai Ni, Hai Huang

**Affiliations:** 1 Department of General Surgery, Hangzhou TCM Hospital Affiliated to Zhejiang Chinese Medical University, #453, Tiyuchang Road, Xihu District, Hangzhou, 310000 Zhejiang Province China; 2 Department of Respiratory and Critical Care Medicine, Hangzhou TCM Hospital Affiliated to Zhejiang Chinese Medical University, #453, Tiyuchang Road, Xihu District, Hangzhou, 310000 Zhejiang Province China

**Keywords:** Stage IB gastric cancer, Chemotherapy, Competing risk analysis

## Abstract

**Background:**

This study aimed to investigate the potential effect of adjuvant chemotherapy in patients diagnosed with stage IB gastric adenocarcinoma (GAC).

**Method:**

A total of 1727 patients were included in the Surveillance, Epidemiology, and End Results (SEER) database from 2004 to 2015 and divided into the chemotherapy and no-chemotherapy groups. Then, the methods of Kaplan-Meier analysis, propensity score matching (PSM), and competing risk analysis were implemented.

**Results:**

After PSM, no significant difference was found in the chemotherapy and no-chemotherapy groups in overall survival (OS) (*p*=0.4) and cancer-specific survival (CSS) (*p*=0.12) in survival curves. The competing risk analysis presented that the 5-year cumulative incidence of cancer-specific death (CSD) was significantly lower in patients receiving chemotherapy (11.5% vs. 20.8%, *p*=0.007), while no significant discrepancy was observed in other causes of death (OCD) in both groups (10.6% vs. 10.9%, *p*=0.474). Multivariable competing risks regression models presented a significant correlation between chemotherapy and CSD (HR, 0.51; 95%CI, 0.31–0.82; *p*=0.007).

**Conclusion:**

The stage IB GAC patients can benefit from adjuvant chemotherapy based on this competing risk analysis.

**Supplementary Information:**

The online version contains supplementary material available at 10.1186/s12957-022-02591-5.

## Introduction

Gastric cancer (GC) is one of the most frequently occurring cancers and the third principal reason for tumor-related death worldwide [1]. And gastric adenocarcinoma (GAC) is the most common subtype of GC [2]. Current postoperative adjuvant therapeutic methods are mainly based on the ACTS-GC trial [3] and the CLASSIC trial [4]. Chemotherapy has been indicated to have a positive effect on postoperative GC patients. However, the trials did not elaborate the role of chemotherapy in stage IB GC (including stage T2N0M0 GC as well as T1a/1bN1M0 GC based on the 8^th^ edition of the American Joint Committee on Cancer (AJCC) tumor-lymph node-metastasis (TNM) stage system). So, unlike stage II–III GC, controversies are still existing regarding the application of chemotherapy for stage IB GC because of the absence of major randomized clinical trials (RCTs).

Based on the evidence provided by these clinical trials, different regional treatment guidelines were constructed. National Comprehensive Cancer Network (NCCN) guideline recommended chemotherapy in high-risk T2N0M0 and T1a/1bN1M0 GC patients who underwent D1 gastrectomy [5]. Besides, the European guideline stated that adjuvant chemotherapy was beneficial for stage T2N0M0 GC and stage T1a/1bN1M0 GC after D2 gastrectomy [6]. On the contrary, the Japanese guideline only recommended a watch-and-wait approach for stage I patients [7]. Therefore, there was no agreement on the benefit of adjuvant chemotherapy in stage IB GC patients.

This retrospective study aimed to use the Surveillance, Epidemiology, and End Results (SEER) database to investigate the potential effect of chemotherapy in stage IB GAC patients.

## Materials and methods

### Data source and patient selection

**Fig. 1:**
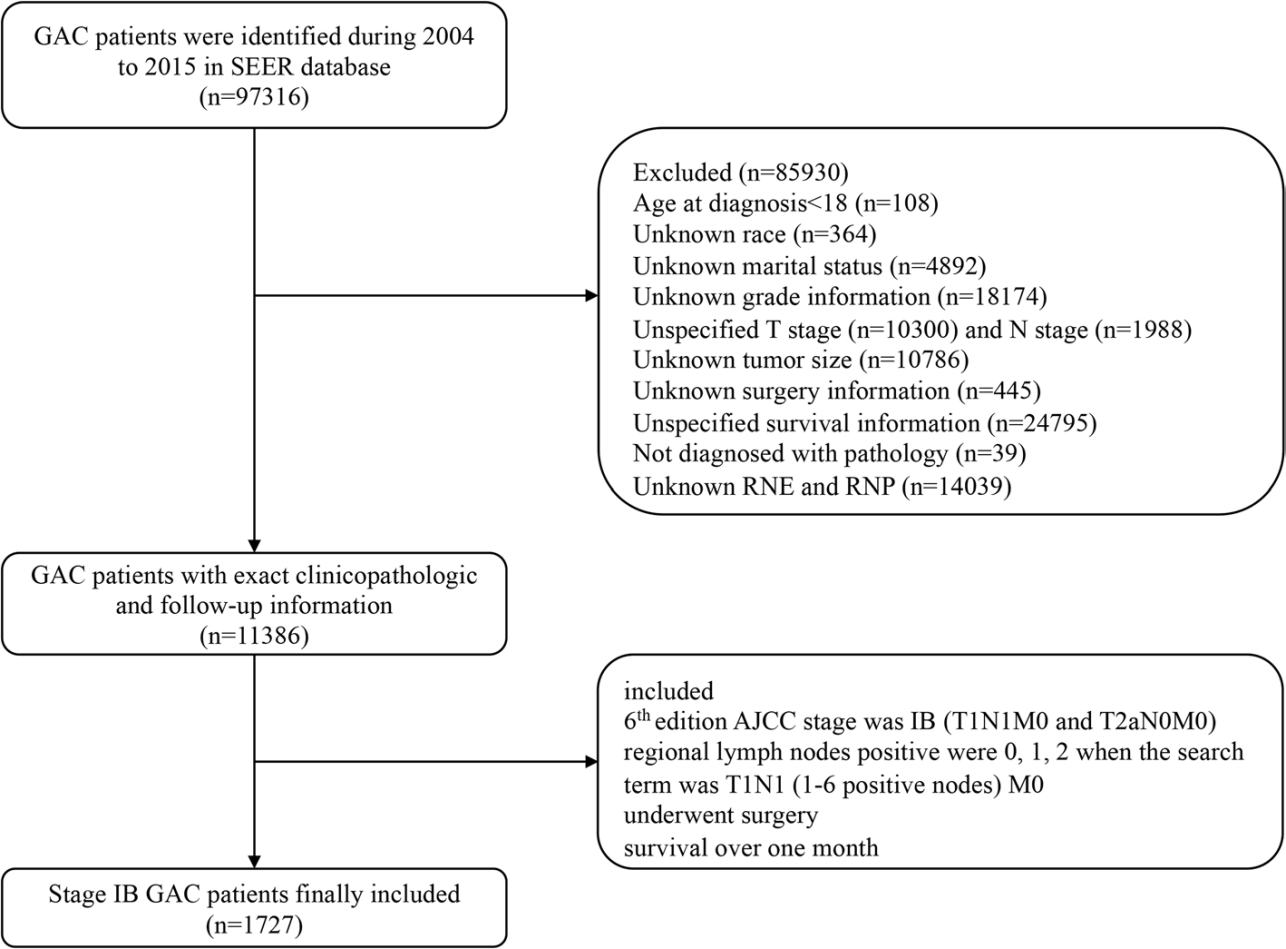
The workflow of the patient selection process

Patients were extracted from the SEER 18 regions database [Incidence-SEER Research Plus data, 18 Registries, Nov 2000 Sub (2000–2018)] using SEER*Sat software (Version 8.3.5). Patients who met the inclusion criteria were identified: (1) age over 18 years; (2) histology ICD-O-3 (International Classification of Diseases for Oncology, 3^rd^ edition) code was adenocarcinoma (8140), mucinous adenocarcinoma (8480), mucin-generating adenocarcinoma (8481), mucinous cyst-adenocarcinoma (8470), signet ring cell carcinoma (8490), papillary adenocarcinoma (8260), tubular adenocarcinoma (8211), adenocarcinoma intestinal type (8144), carcinoma—diffuse type (8145), and adenocarcinoma with mixed subtype (8255); (3) patients who underwent surgery without distant metastasis; (4) patients who survived over one month; and (5) the 6^th^ edition AJCC stage was IB and the 6^th^ edition AJCC TNM were T1N1M0 or T2aN0M0. The exclusion criteria were as follows: (1) regional positive lymph nodes were 3, 4, 5, and 6 when the search term was T1N1 (1–6 positive nodes) M0, since all the staging data were converted to adapt to the 8^th^ edition of AJCC TNM staging system to obtain an adequate follow-up duration; (2) patients with incomplete demographic, clinic-pathological, treatment, and follow-up information were excluded. Ultimately, 1727 cases were included in the study. The detailed patient selection workflow is shown in Fig. 1.

### Clinicopathological variables

Demographic data (age, year at diagnosis, gender, race, marital status), grade, T stage, primary site, tumor size, regional nodes examined (RNE), regional nodes positive (RNP), radiation, chemotherapy, and prognostic information were collected for each patient. All patients were split into chemotherapy and no-chemotherapy cohorts. And the race was classified into white and nonwhite.

### Statistical analysis

Differences in the baseline clinicopathological variables were tested using *t*-test and chi-square test. Kaplan-Meier analyses were used to compare the overall survival (OS) and cancer-specific survival (CSS). To accurately evaluate the influence of competing risk factors, patients were divided into three endpoints of interest: alive, cancer-specific death (CSD), and other causes of death (OCD). Cumulative incidence function and Fine and Grey’s proportional subdistribution hazard model were further used to assess the effects of chemotherapy in stage IB GAC cases with the R package “cmprsk” [8].

The propensity score matching (PSM) method was performed to minimize the heterogeneity and facilitate matching patients in two cohorts [9]. All variables were used to generate a propensity score by logistic regression. Patients in both groups were matched using the one-to-one nearest-neighbor algorithm on the logit scale. The caliper was set at 0.01. We used standardized difference (SD) to present the change of variables before and after PSM. SD ≤0.1 indicated ideal balances in the baseline parameters [10]. After PSM, SD in most variables was less than 0.1, which showed good balancing performance (Fig. S1).

All statistical analyses and visualization were based on R software, version 4.0.3 (http://www.r-project.org). A two-tailed *P*<0.05 was indicated to be statistically significant.

## Results

### Clinicopathological characteristics

A total of 1727 patients with stage IB GAC were finally recruited in the SEER database from 2004 to 2015. Of these patients, 610 patients had received chemotherapy, while the other 1117 patients had not been treated with chemotherapy. There were significant dissimilarities among the two cohorts in the characteristics, including age, gender, marital status, grade, T stage, primary site, RNE, RNP, and radiation (all *p*<0.05). The patients underwent chemotherapy tended to present higher proportion of male (67.9% vs. 61.1%), married status (67.7% vs. 61.3%), III/IV grade (60.3% vs. 51.7%), T1 stage (44.6% vs. 20.7%), and regional node examined more than 16 (44.4% vs. 38.0%). The no-chemotherapy group presented more percentage when the regional node-positive was zero (79.7% vs. 58.5%).

In view of unmatched parameters between the two cohorts, we performed 1:1 PSM to reduce the influence of potential confounders. Ultimately, 470 patients were separated into group chemotherapy (*n*=235) and group no-chemotherapy (*n*=235). The baseline characteristics before and after PSM are presented in Tables 1 and 2.

**Table 1:** The descriptive characteristics of stage IB GAC patients before PSM

Characteristics	Before PSM			*P* value
	All	No Chemo	Chemo	
	1727	1117	610	
Age	68.7 (12.3)	72.1 (11.4)	62.4 (11.2)	<0.001
Year				<0.001
2004–2007	634 (36.7%)	446 (39.9%)	188 (30.8%)	
2008–2011	553 (32.0%)	373 (33.4%)	180 (29.5%)	
2012–2015	540 (31.3%)	298 (26.7%)	242 (39.7%)	
Gender				0.006
Female	631 (36.5%)	435 (38.9%)	196 (32.1%)	
Male	1096 (63.5%)	682 (61.1%)	414 (67.9%)	
Race				0.202
White	1128 (65.3%)	717 (64.2%)	411 (67.4%)	
Non-White	599 (34.7%)	400 (35.8%)	199 (32.6%)	
Marital status				0.011
Married	1098 (63.6%)	685 (61.3%)	413 (67.7%)	
Unmarried	629 (36.4%)	432 (38.7%)	197 (32.3%)	
Grade				0.001
I/II	782 (45.3%)	540 (48.3%)	242 (39.7%)	
III/IV	945 (54.7%)	577 (51.7%)	368 (60.3%)	
T stage				<0.001
T1	503 (29.1%)	231 (20.7%)	272 (44.6%)	
T2a	1224 (70.9%)	886 (79.3%)	338 (55.4%)	
Primary site				<0.001
Cardia/fundus	534 (30.9%)	291 (26.1%)	243 (39.8%)	
Greater curvature/lesser curvature/body	456 (26.4%)	297 (26.6%)	159 (26.1%)	
Others	178 (10.3%)	125 (11.2%)	53 (8.7%)	
Pylorus/antrum	559 (32.4%)	404 (36.2%)	155 (25.4%)	
Tumor size				0.054
≤2cm	532 (30.8%)	322 (28.8%)	210 (34.4%)	
≤5cm	902 (52.2%)	599 (53.6%)	303 (49.7%)	
>5cm	293 (17.0%)	196 (17.5%)	97 (15.9%)	
RNE				0.011
16	696 (40.3%)	425 (38.0%)	271 (44.4%)	
1–15	1031 (59.7%)	692 (62.0%)	339 (55.6%)	
RNP				<0.001
0	1247 (72.2%)	890 (79.7%)	357 (58.5%)	
1	329 (19.1%)	173 (15.5%)	156 (25.6%)	
2	151 (8.7%)	54 (4.8%)	97 (15.9%)	
Radiation				<0.001
None	1304 (75.5%)	1084 (97.0%)	220 (36.1%)	
Yes	423 (24.5%)	33 (3.0%)	390 (63.9%)

**Table 2:** The descriptive characteristics of stage IB GAC patients after PSM

Characteristics	After PSM			*P* value
	All	No Chemo	Chemo	
	470	235	235	
Age	63.4 (11.3)	63.5 (11.4)	63.2 (11.1)	0.743
Year				0.819
2004–2007	122 (26.0%)	64 (27.2%)	58 (24.7%)	
2008–2011	138 (29.4%)	68 (28.9%)	70 (29.8%)	
2012–2015	210 (44.7%)	103 (43.8%)	107 (45.5%)	
Gender				1
Female	157 (33.4%)	79 (33.6%)	78 (33.2%)	
Male	313 (66.6%)	156 (66.4%)	157 (66.8%)	
Race				0.386
White	302 (64.3%)	146 (62.1%)	156 (66.4%)	
Non-White	168 (35.7%)	89 (37.9%)	79 (33.6%)	
Marital status				1
Married	313 (66.6%)	157 (66.8%)	156 (66.4%)	
Unmarried	157 (33.4%)	78 (33.2%)	79 (33.6%)	
Grade				0.779
I/II	194 (41.3%)	95 (40.4%)	99 (42.1%)	
III/IV	276 (58.7%)	140 (59.6%)	136 (57.9%)	
T stage				0.292
T1	172 (36.6%)	80 (34.0%)	92 (39.1%)	
T2a	298 (63.4%)	155 (66.0%)	143 (60.9%)	
Primary site				0.799
Cardia/fundus	166 (35.3%)	78 (33.2%)	88 (37.4%)	
Greater curvature/lesser curvature/body	131 (27.9%)	68 (28.9%)	63 (26.8%)	
Others	49 (10.4%)	26 (11.1%)	23 (9.8%)	
Pylorus/antrum	124 (26.4%)	63 (26.8%)	61 (26.0%)	
Tumor size				0.868
≤2cm	158 (33.6%)	79 (33.6%)	79 (33.6%)	
≤5cm	238 (50.6%)	121 (51.5%)	117 (49.8%)	
>5cm	74 (15.7%)	35 (14.9%)	39 (16.6%)	
RNE				0.782
≥16	240 (51.1%)	122 (51.9%)	118 (50.2%)	
1–15	230 (48.9%)	113 (48.1%)	117 (49.8%)	
RNP				0.263
0	309 (65.7%)	159 (67.7%)	150 (63.8%)	
1	110 (23.4%)	56 (23.8%)	54 (23.0%)	
2	51 (10.9%)	20 (8.5%)	31 (13.2%)	
Radiation				1
None	404 (86.0%)	202 (86.0%)	202 (86.0%)	
Yes	66 (14.0%)	33 (14.0%)	33 (14.0%)	

### Survival analysis

**Fig. 2:**
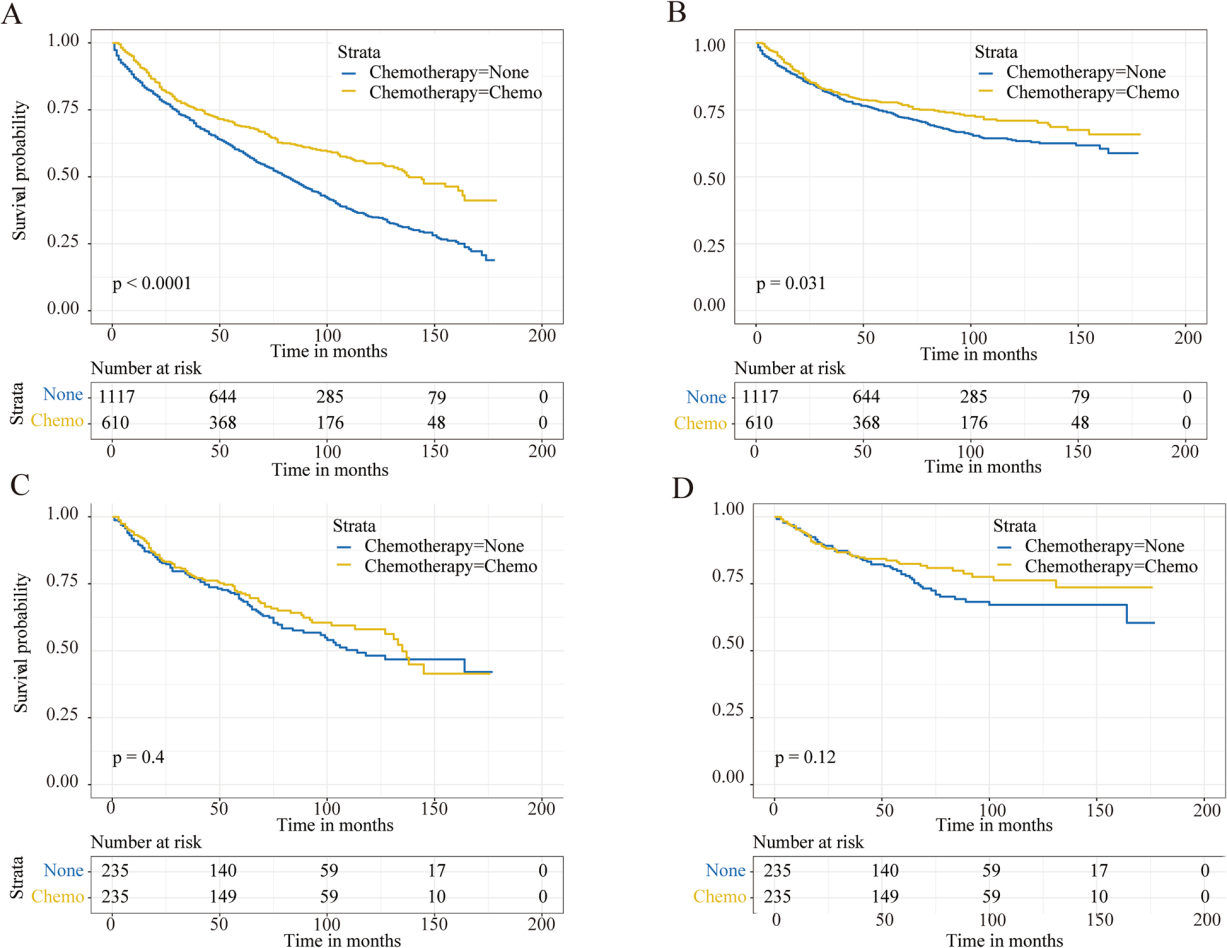
OS and CSS of the stage IB GAC patients for the chemotherapy and no-chemotherapy cohorts. OS (**A**) and CSS (**B**) curves before PSM. OS (**C**) and CSS (**D**) curves after PSM

**Fig. 3:**
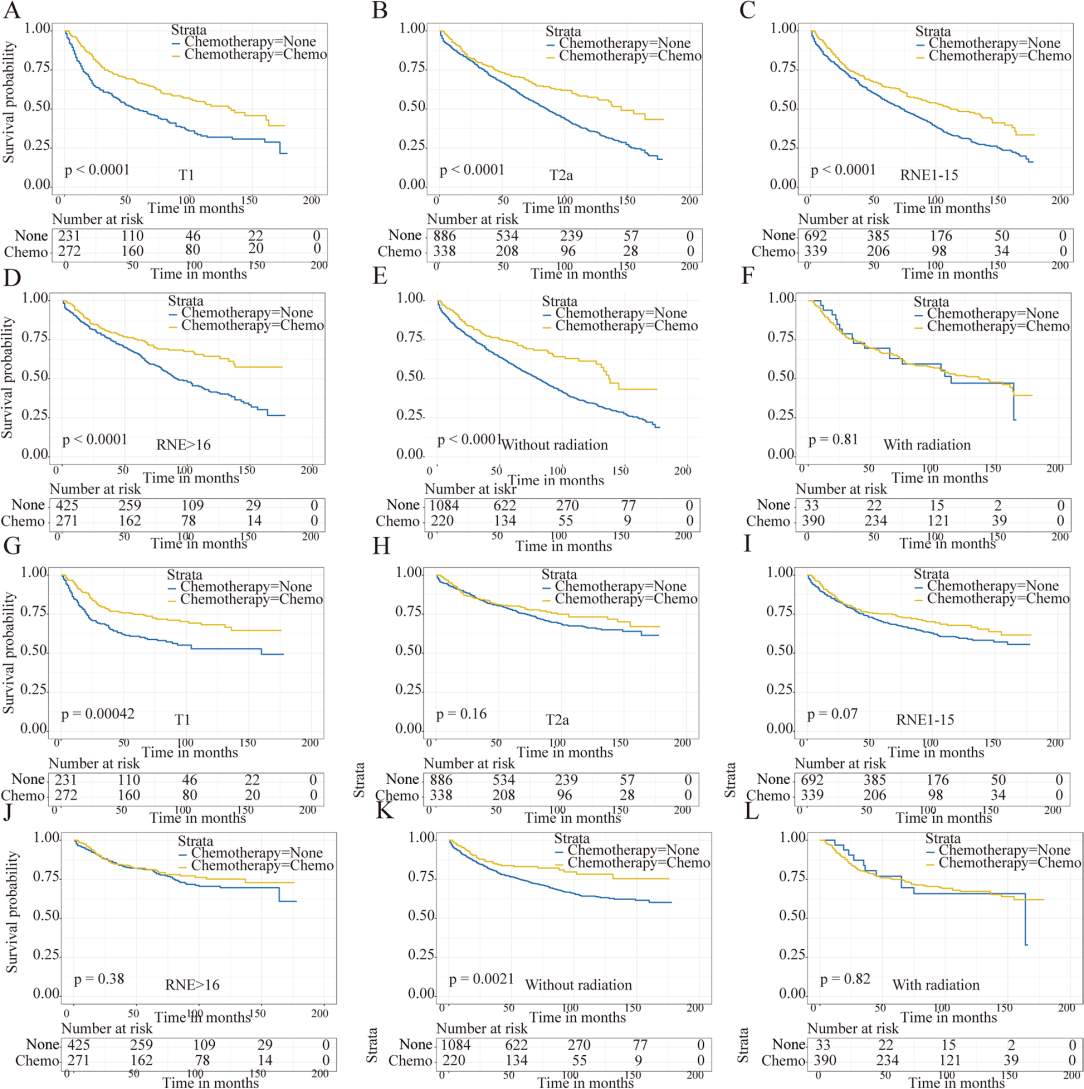
Survival curves of the stage IB GAC patients for the chemotherapy and no-chemotherapy cohorts in different subgroups before PSM. OS (**A**) and CSS (**G**) in the T1 cohort. OS (**B**) and CSS (**H**) in the T2a cohort. OS (**C**) and CSS (**I**) in the RNE1-15 cohort. OS (**D**) and CSS (**J**) in RNE>16 cohort. OS (**E**) and CSS (K) in without radiation cohort. OS (**F**) and CSS (**L**) in with radiation cohort

The patients who underwent chemotherapy presented better survival compared with the no-chemotherapy cohort in OS (5-year survival: 68.8% vs. 59.5%, *p*<0.001) and CSS (5-year survival: 77.8% vs. 74.4%, *p*=0.031) (Fig. 2A, B). Then, the subgroup analyses based on T stage, RNE, and radiation were performed, presenting a longer 5-year OS in patients receiving chemotherapy except in the radiation subgroup (Fig. 3A–F). But significantly higher 5-year CSS in the chemotherapy cohort was only found in the T1 and no-radiation subgroup (Fig. 3 G,K). Besides, the univariate Cox analyses revealed that chemotherapy was associated with better OS (HR, 0.63; 95%CI, 0.55–0.73; *p*<0.001) and CSS (HR, 0.81; 95%CI, 0.67–0.98; *p*=0.03), which was similar with the Kaplan-Meier analyses.

**Fig. 4:**
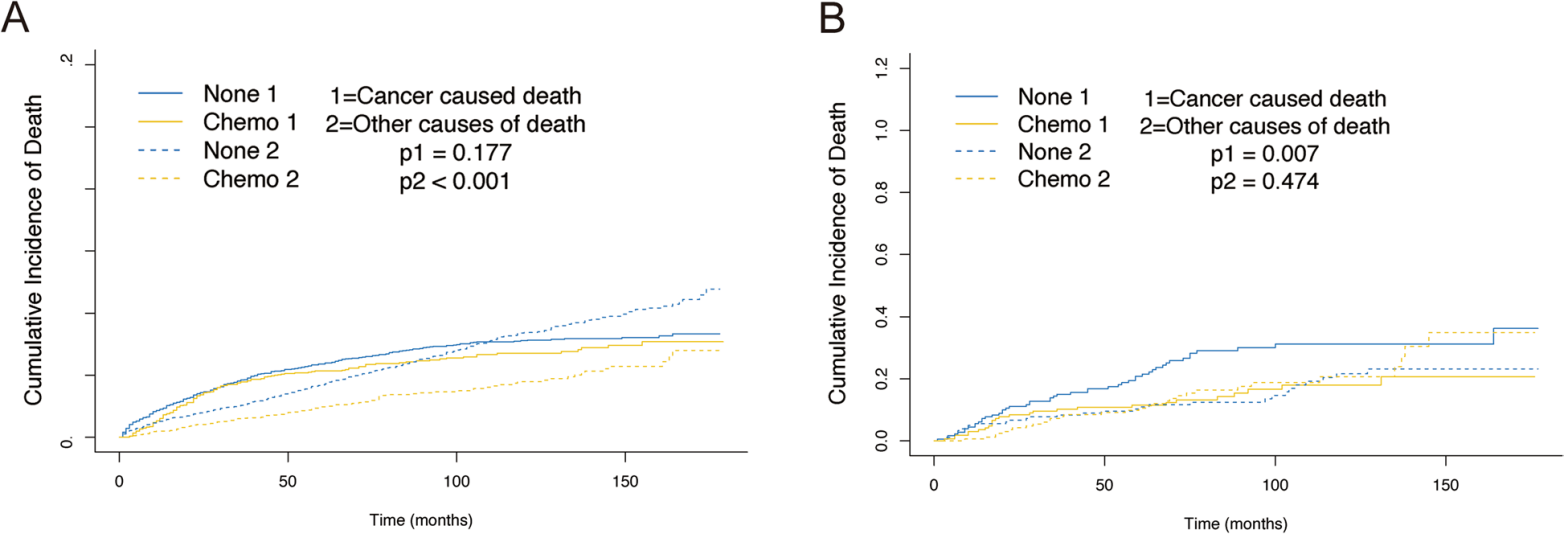
Cumulative incidence curves describing cancer-specific deaths and deaths from other causes before and after PSM. **A** Cumulative incidence curves before PSM. **B** Cumulative incidence curves after PSM

**Fig. 5:**
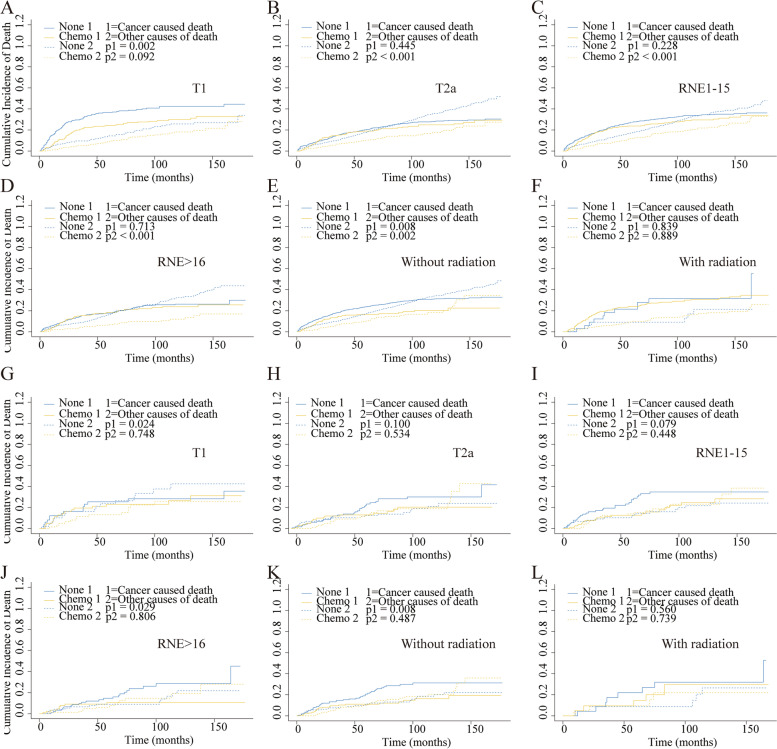
Cumulative incidence curves for the stage IB GAC patients according to chemotherapy in different subgroups before and after PSM. T1 (**A**), T2a (**B**), RNE1-15 (**C**), RNE>16 (**D**), without radiation (**E**), and with radiation (**F**) cohorts before PSM. T1 (**G**), T2a (**H**), RNE1-15 (**I**), RNE>16 (**J**), without radiation (**K**), and with radiation (**L**) cohorts after PSM

Considering the competing risk factors, cumulative incidence plots were further constructed, presenting similar 5-year cumulative incidence of CSD in two cohorts (21.4% vs. 23.6%, *p*=0.177) and a significantly lower 5-year cumulative incidence of OCD among patients who underwent chemotherapy (9.8% vs. 16.9%, *p*<0.001) (Fig. 4A, Table 3). Subsequently, the subgroups analysis for T stage, RNE, and radiation were performed. The results showed that the patients without chemotherapy suffered higher CSD in the T1 stage and no-radiation cohort (Fig. 5A, E). But no similar outcomes were found referring to other subgroups (Fig. 5B–D, F). In the multivariable competing risks regression analysis, a significant correlation was found between chemotherapy and CSD (HR, 0.71; 95%CI, 0.53–0.96; *p*=0.028) (Table 4).

**Table 3:** The effect of chemotherapy for stage IB GAC patients by cumulative incidence function analysis before and after PSM

	5-year CID of cancer	*P* value	5-year CID of other causes	*P* Value
Before PSM				
No_Chemo	0.236	0.177	0.169	<0.001
Chemo	0.214		0.098	
After PSM				
No_Chemo	0.208	0.007	0.109	0.474
Chemo	0.115		0.106	

*CID* Cumulative incidences of death

**Table 4:** The results of multivariate subdistribution hazard model for stage IB GAC patients

Characteristics	Before PSM			After PSM		
	HR	95%CI	*p* value	HR	95%CI	*p* value
Age	1.02	1.01–1.03	<0.001	1.01	0.99–1.04	0.281
Year						
2004–2007	Reference			Reference		
2008–2011	0.74	0.60–0.92	0.006	0.56	0.31–1.04	0.066
2012–2015	0.71	0.55–0.89	0.004	0.86	0.47–1.56	0.611
Gender						
Female	Reference			Reference		
Male	1.32	1.06–1.65	0.013	1.92	1.04–3.54	0.037
Race						
White	Reference			Reference		
Non-White	0.99	0.81–1.22	0.941	1.04	0.59–1.84	0.881
Marital status						
Married	Reference			Reference		
Unmarried	1.14	0.93–1.40	0.222	1.16	0.65–2.07	0.621
Grade						
I/II	Reference			Reference		
III/IV	1.11	0.92–1.33	0.271	1.02	0.62–1.73	0.941
T stage						
T1	Reference			Reference		
T2a	0.81	0.39–1.66	0.552	2.18	0.53–8.94	0.282
Primary site						
Cardia/fundus	Reference			Reference		
Greater curvature/lesser curvature/body	0.48	0.38–0.62	<0.001	0.58	0.32–1.05	0.072
Others	0.52	0.37–0.73	<0.001	0.25	0.10–0.67	0.006
Pylorus/antrum	0.4	0.31–0.51	<0.001	0.27	0.12–0.6	0.001
Tumor size						
≤2cm	Reference			Reference		
≤5cm	1.06	0.86–1.32	0.611	1.51	0.86–2.64	0.152
>5cm	1.02	0.75–1.39	0.892	1.56	0.71–3.44	0.271
RNE						
≥16	Reference			Reference		
1–15	1.33	1.09–1.62	0.004	1.63	1.02–2.63	0.043
RNP						
0	Reference			Reference		
1	1.17	0.56–2.47	0.683	2.78	0.65–11.93	0.171
2	1.57	0.72–3.39	0.251	1.94	0.35–10.67	0.442
Radiation						
None	Reference			Reference		
Yes	1.29	0.95–1.77	0.113	1.54	0.82–2.97	0.201
Chemotherapy						
None	Reference			Reference		
Yes	0.71	0.53–0.96	0.028	0.51	0.31-0.82	0.007

After 1:1 PSM, a significant difference was found for the 5-year cumulative incidences of CSD between the two groups (11.5% vs. 20.8%, *p*=0.007). But no similar result was shown for the 5-year cumulative incidence of OCD between the two groups (10.6% vs. 10.9%, *p*=0.474) (Fig. 4B, Table 3). Then the subgroups analyses were performed again, indicating a significant correlation between chemotherapy and CSD in subgroups of T1 stage, RNE>16, and no-radiation cohorts (Fig. 5G, J, K). And there was a significant relationship between chemotherapy and CSD in the multivariate competing risks regression analysis (HR, 0.51; 95%CI, 0.31–0.82; *p*=0.007) (Table 4).

### Age-stratified analysis

Considering the role of age in the application of chemotherapy in stage IB GAC patients, we performed age-stratified analyses based on the data after PSM. The cutoff age was set to 70 years old. In the cohort of patients older than 70 years, the results did not present a significant difference in the 5-year cumulative incidence of CSD in both cohorts (15.6% vs. 26.4 %, *p*=0.085) (Table S1). Then in the cohort of patients younger than 70 years, a significant difference was found in the 5-year cumulative incidences of CSD between the two groups (9.6% vs. 17.6%, *p*=0.021) (Table S2). The results indicated that compared with young patients, old patients might not benefit from chemotherapy in stage IB GAC patients. The further subgroup analyses confirmed this point (all *p*>0.05) (Fig. S2).

## Discussion

Several RCTs have been conducted to investigate the effect of chemotherapy in GC patients. A SWOG-directed INT-0116 trial reported superior OS and relapse-free survival in stage II/III GC patients who underwent adjuvant chemoradiotherapy [11]. MAGIC [12], ACTS-GC [13], and CLASSIC [4] trials also presented a benefit in prognosis when performing adjuvant chemotherapy within the majority of high-stage cases. Nevertheless, whether all stages of GC (especially for stage IB GC) that underwent chemotherapy could gain benefit was not precisely explained. Besides, the administration of chemotherapy among stage IB GC remained controversial in different regional guidelines [5, 7, 14]. The European consensus stated that adjuvant chemotherapy was beneficial for stage T2N0M0 GC and stage T1a/1bN1M0 GC after D2 gastrectomy [6]. On the contrary, the Japanese guideline only recommended a watch-and-wait-approach for stage I patients [7].

Many retrospective studies have concentrated on the stage IB GC for the absence of relevant RCTs. Seyedin et al. examined adjuvant therapy-related results in stage IB GC patients, suggesting that adjuvant therapy can improve survival compared with the surgery alone cohort [15]. Besides, one recent research based on National Cancer Database revealed a significantly lower risk-adjusted mortality rate among stage IB patients who receive adjuvant chemoradiotherapy [16]. However, a Korean study, which included 738 stage IB GC cases, indicated no benefit from adjuvant chemotherapy on disease-free survival and tumor recurrence among these patients, which supported the recommendations of Japanese guideline [17]. And in another retrospective study recruiting over 2000 GC patients who underwent gastrectomy, the authors revealed that the subset of cases with early-stage (stage I-T1/T2, N0, or T1/N1) might benefit from adjuvant chemotherapy [18]. The limited population of stage IB GC patients and various endpoints used in former research have hindered the presentation of the real effects of chemotherapy. To the best of our knowledge, this is the first research focusing on the effect of chemotherapy in stage IB GAC patients based on the competing risk analysis and to give evidence supporting chemotherapy in these specific patients.

OS and CSS are considered the most critical endpoint in cancer research to investigate whether patients can benefit from therapy. The Kaplan-Meier and Cox proportional hazard regression models are widely used in survival analysis, clarifying the relationship between risk factors and clinical outcomes. In this study, the 5-year OS rates of the patients were 68.8% in the chemotherapy group and 59.5% in the no-chemotherapy group, which were lower than the patients with stage II disease in the ACTSGC trial (84.2% in the S-1 group and 71.3% in the surgery group) [13]. Several reasons might explain this seemingly abnormal result. In the ACTSGC trial, only patients with adequate organ function were enrolled, which might partly explain the better survival. Secondly, all patients in the ACTSGC trial underwent high-quality D2 gastrectomy in Japan, whereas D0 or D1 gastrectomy remained the standard procedure in the USA at the same time. And a 15-year follow-up study presented that the GC-related death rates were significantly lower in the D2 gastrectomy group [19]. The present study identified 1727 post-gastrectomy stage IB GAC cases in the SEER database, indicating that patients who underwent adjuvant chemotherapy did not present better OS or CSS after PSM. However, OCD was not considered in these survival analyses, which might hinder the observation of actual situations. Especially in stage IB GAC patients who experience relatively long 5-year survival rates [20], patients tended to die of other competing causes, such as other malignancy and cardiovascular diseases. We performed a competing risk analysis for the objective presence of the competing risk factors that cannot be ignored and censored. After PSM, the results presented that the 5-year cumulative incidence of CSD was significantly lower in patients receiving chemotherapy (11.5% vs. 20.8%, *p*=0.007), while no significant discrepancy was observed in OCD in both groups (10.6% vs. 10.9%, *p*=0.474). And multivariable competing risks regression models presented a significant correlation between chemotherapy and CSD (HR, 0.51; 95%CI, 0.31–0.82; *p*=0.007). These outcomes demonstrated that patients in two cohorts suffered similar non-cancer-specific death, but patients who underwent chemotherapy presented significantly lower cancer-specific death.

Interestingly, not all stage IB GAC patients were benefited from chemotherapy in the subgroup analysis (Fig. 5). The results did not indicate a significant correlation between chemotherapy and CSD in the subgroups of T2a stage, RNE1-15, and radiation cohorts. However, the *p*-value was nearly significant in the T2a stage and RNE1-15 subgroup, the failure to reach statistical significance might be partly attributed to the much smaller size of the PSM groups (235 patients). In the radiation subgroup, the patients who underwent chemotherapy did not improve CSD, perhaps because stage IB GAC was highly curable by surgery alone, and the toxicity from chemoradiotherapy outweighed the benefit [21]. Considering the role of age in the application of chemotherapy in stage IB GAC patients, we also performed age-stratified analyses. The results indicated that compared with young patients, old patients might not benefit from chemotherapy in stage IB GAC patients. The reason could be that elderly patients tend to suffer from OCD. Further identification of the specific population who might benefit from chemotherapy was required.

### Limitation

The research provided convincing evidence for the application of chemotherapy in stage IB GAC patients. However, several limitations that existed in the current study should raise attention. Firstly, the absence of detailed chemotherapy information caused the dilemma of comparing the influence of different chemotherapy agents. Secondly, the database did not include information on recurrence, which hindered further contrast with previous studies. Also, this was a retrospective analysis, so selection bias was inevitably brought in. Despite these limitations, the large number of cases could provide novel insights on the chemotherapy in stage IB GAC patients.

## Conclusion

Using competing risk analysis and 1:1 PSM analysis, we demonstrated that stage IB GAC patients suffered lower CSD when receiving adjuvant chemotherapy. The results give strong evidence supporting chemotherapy in these specific patients. Further research and RCTs are required to validate the conclusion.

## Supplementary Information

**Additional file 1: Figure S1.** The mean difference between the two cohorts.

**Additional file 2: Figure S2.** Cumulative incidence curves for the old stage IB GAC patients according to chemotherapy in different subgroups after PSM. T1 (A), T2a (B), RNE1-15 (C), RNE>16 (D), without radiation (E) and with radiation (F) cohorts.

**Additional file 3: Table S1.** The effect of chemotherapy for old stage IB GAC patients by cumulative incidence function analysis before and after PSM.

**Additional file 4: Table S2.** The effect of chemotherapy for young stage IB GAC patients by cumulative incidence function analysis before and after PSM.

## Data Availability

The datasets generated and analyzed during the current study are available in the SEER database (https://seer.cancer.gov/) and from the corresponding authors upon reasonable request.
