# Supplementary figures and images for: The role of chemotherapy in patients with stage IB gastric adenocarcinoma: a real-world competing risk analysis
Source: World J Surg Oncol. 2022 Apr 17;20:123. doi: 10.1186/s12957-022-02591-5 (PMC9013453; doi:10.1186/s12957-022-02591-5)

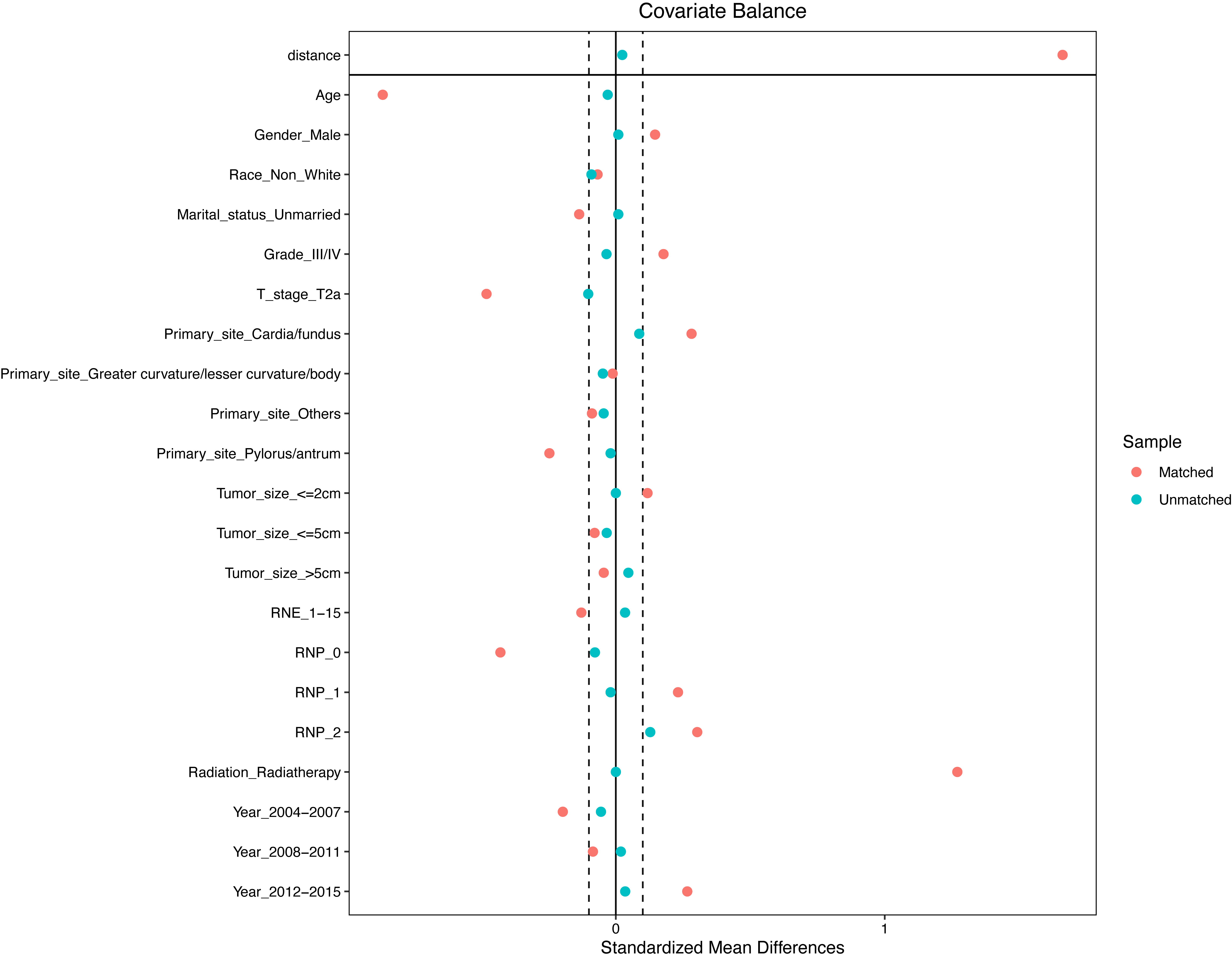

Supplement: Supplementary file 1 — Additional file 1: Figure S1. The mean difference between the two cohorts. [file 12957_2022_2591_MOESM1_ESM.tif]

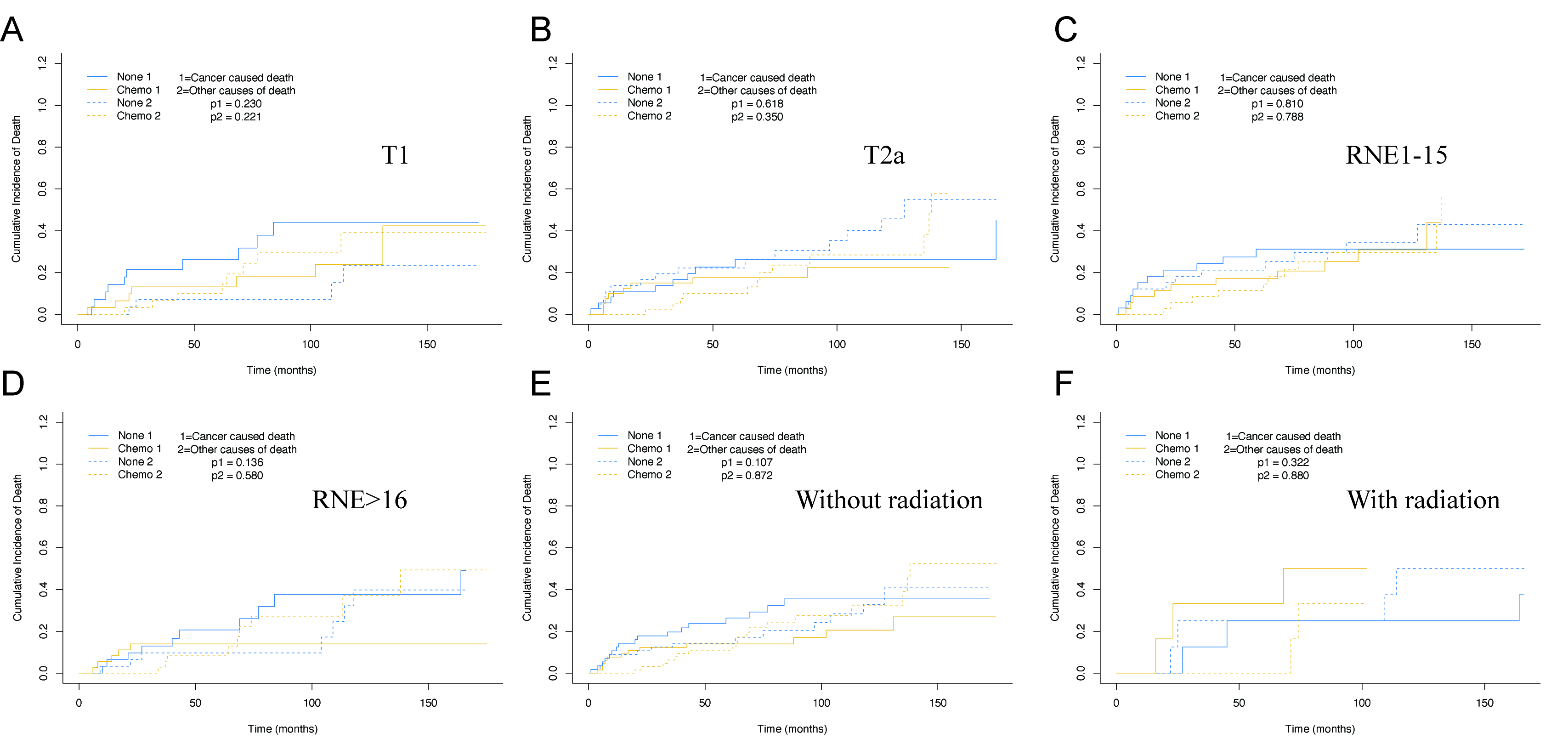

Supplement: Supplementary file 2 — Additional file 2: Figure S2. Cumulative incidence curves for the old stage IB GAC patients according to chemotherapy in different subgroups after PSM. T1 (A), T2a (B), RNE1-15 (C), RNE>16 (D), without radiation (E) and with radiation (F) cohorts. [file 12957_2022_2591_MOESM2_ESM.tif]
