# Supplementary material for: The role of chemotherapy in patients with stage IB gastric adenocarcinoma: a real-world competing risk analysis
Source: World J Surg Oncol. 2022 Apr 17;20:123. doi: 10.1186/s12957-022-02591-5 (PMC9013453; doi:10.1186/s12957-022-02591-5)
Supplement: Supplementary file 4 — Additional file 4: Table S2. The effect of chemotherapy for young stage IB GAC patients by cumulative incidence function analysis before and after PSM. [file 12957_2022_2591_MOESM4_ESM.docx]

| Table S2. The effect of chemotherapy for young stage IB GAC patients by cumulative incidence function analysis before and after PSM | | | | |
| --- | --- | --- | --- | --- |
|  | 5-year CID of cancer | P value | 5-year CID of other causes | P Value |
| Before PSM |  |  |  |  |
| No Chemo | 0.187 | 0.138 | 0.112 | 0.017 |
| Chemo | 0.133 |  | 0.091 |  |
| After PSM |  |  |  |  |
| No Chemo | 0.176 | 0.021 | 0.094 | 0.539 |
| Chemo | 0.096 |  | 0.083 |  |
| CID, cumulative incidences of death | |  |  |  |
